# Our Publishers

**Published:** 1873-02

**Authors:** 


### Editorial.

***Our Publishers***

Are "home again" in their new quarters, but the old location before the fire—*in statu quo ante ignem*—and we were about preparing an account of the same, when we happened upon this extract from the correspondence of the *Weekly Trade Circular* of New York, which we reproduce and indorse, and to which we add "the half has not been told," and if, then, anybody is incredulous, let him "come and see." Chicago (our Publishers and Printers inclusive) has seen fiery tribulations, compared to which ancient Job's were a bagatelle, but like him its latter days are getting to be its best days.

"Messrs. Keen, Cooke & Co. are now taking the lead here in the general book trade. This is due, in a large measure, to their wise policy in providing themselves with spacious and convenient quarters immediately after the fire, and thus having the stock room, and will to do all the business that came to them at a time when the other dealers were unfortunately crowded into narrow and confined space. The result of the fire, therefore, has been rather to widely extend and stimulate their business than to cripple it. The retirement of Messrs. S. C. Griggs & Co. from the trade has also, no doubt, had its influence. Messrs. Keen, Cooke & Co. have just removed from their temporary quarters, corner Washington street and Wabash avenue, to their old stand, 113 and 115 State street, but into a new building, and, as compared with the old one, much more elegant and convenient in every respect. The building is one of the finest marble fronts in the city, and is known as the Williams & Ferry Building. It was planned with special reference to the book trade, and the five stories are shared by Messrs. Keen, Cooke & Co. with Messrs. A. S. Barnes & Co. The retail department, on the first floor, occupies space 50 by 150 feet, and is beautifully and systematically arranged, the shelves on either side being apportioned according to their respective importance to the various American and foreign publishers, and the books therein being alphabetically arranged so that every clerk can lay his hand on any book even in the dark. Busts of prominent authors, ancient and modern, appropriately adorn and dignify these separate alcoves. The room is lighted with reflectors *a la* Ball & Black's in New York, and all other appurtenances are of like appropriateness and elegance. The basement is 50 by 200, and exclusively devoted to school books, stationery, inks, etc. The second story is used for small stationery and as a packing room. All the several departments are connected and communicated with by a steam elevator as well as by spacious stairways.

"Altogether the establishment presents a very attractive appearance, whether to the lover or purchaser of books, and excels, in outward appearance at least, anything we have ever had in Chicago in the way of a book store."

